# Predictors of Childhood Anxiety: A Population-Based Cohort Study

**DOI:** 10.1371/journal.pone.0129339

**Published:** 2015-07-09

**Authors:** Dawn Kingston, Maureen Heaman, Marni Brownell, Okechukwu Ekuma

**Affiliations:** 1 Faculties of Nursing and Medicine (Psychiatry, Obstetrics and Gynecology), University of Alberta, Edmonton, Alberta, Canada; 2 Faculty of Nursing, University of Manitoba, Winnipeg, Manitoba, Canada; 3 Faculty of Medicine, Manitoba Centre for Health Policy, University of Manitoba, Winnipeg, Manitoba, Canada; University of Illinois-Urbana Champaign, UNITED STATES

## Abstract

**Background:**

Few studies have explored predictors of early childhood anxiety.

**Objective:**

To determine the prenatal, postnatal, and early life predictors of childhood anxiety by age 5.

**Methods:**

Population-based, provincial administrative data (N = 19,316) from Manitoba, Canada were used to determine the association between demographic, obstetrical, psychosocial, medical, behavioral, and infant factors on childhood anxiety.

**Results:**

Risk factors for childhood anxiety by age 5 included maternal psychological distress from birth to 12 months and 13 months to 5 years post-delivery and an infant 5-minute Apgar score of ≤7. Factors associated with decreased risk included maternal age < 20 years, multiparity, and preterm birth.

**Conclusion:**

Identifying predictors of childhood anxiety is a key step to early detection and prevention. Maternal psychological distress is an early, modifiable risk factor. Future research should aim to disentangle early life influences on childhood anxiety occurring in the prenatal, postnatal, and early childhood periods.

## Introduction

Mental health disorders are the leading health problems in children [1]. With lifetime prevalence rates of 15%-20% [2], anxiety represents the most common childhood psychopathology [3,4]. Childhood anxiety increases the risk for anxiety onset later in life [2], and is associated with long-term consequences related to school achievement and development [5]. Rates of DSM anxiety disorders in children 1 to 6 years of age range 3.0 to 6.6% [4,6], while population-based rates based on screening tools for the same age group assessed in community-based settings are higher at 11.9% [4]. It is concerning that in the National Survey of Child Health, 2 to 5 year olds had the lowest mental health service utilization rate (42.2%) of all age groups (http://www.childhealthdata.org/learn/NSCH). Additionally, in Canada, the prevalence of children with at least one anxiolytic prescription increased significantly from 2000/01 (5.0/1000) to 2005/06 (6.1/1000) [7], with the greatest increases observed among 1 to 4 year olds. Together, this information suggests that children under 5 years may possess unique risks for anxiety, yet are understudied [8]. However, little is known about the risk factors for anxiety in young children [9,10]. Most research to date has focused on factors associated with anxiety onset in pre-adolescents and adolescents, despite emerging evidence that supports the need to assess and intervene earlier in childhood [11]. Furthermore, existing research is limited by small samples and the inclusion of few predictors, predominantly occurring beyond the perinatal period (e.g., parenting, child temperament [12,13]). As such, this research does not reflect current directions in developmental neurobiology that link prenatal and early postnatal influences to adverse health and developmental outcomes [14]. Thus, existing research provides little guidance regarding prenatal and postnatal predictors of the risk for child anxiety that may facilitate early detection, prevention, and intervention among children most in need. The purpose of this study was to determine the prenatal, postnatal, and early life predictors of childhood anxiety by age 5.

## Methods

### Study Design, Setting and Inclusion Criteria

This population-based cohort study was conducted using data from the Population Health Research Data Repository at the Manitoba Centre for Health Policy (MCHP), University of Manitoba. Women were included in the study if they: 1) delivered a live, singleton infant from January 1, 2003 to December 31, 2004; 2) had their child living with them at age 5; and 3) had a postnatal risk screen completed for the Families First program. In the Families First Program, public health nurses conduct home visits with all new mothers in Manitoba (excluding First Nation mothers living on the reserve) to screen them for social risk factors and facilitate referral to appropriate services. Eighty to 85% of all new mothers in Manitoba are screened, and 17% of those meet eligibility for the Program. In this study, we included all new mothers that had a Families First screen in order to capture data on social risk factors that are not available in the other administrative databases.

### Data Sources and Variables

We used provincial, de-identified administrative databases linked with a scrambled Personal Health Identification Number, including Hospital Discharge Abstracts (all hospitalizations), Medical Claims file (physician visits), Prescription Drug Use files (all outpatient prescription medications), Vital Statistics, Population Registry (demographics), Manitoba Family Services (i.e., social services including child welfare services and income assistance), and Healthy Child Manitoba (Families First screening). A detailed description of the databases can be found online at http://www.umanitoba.ca/faculties/medicine/units/mchp/resources/concept_dictionary.html.

### Definitions

The primary outcome was childhood anxiety by age 5. Childhood anxiety was measured using a combination of hospitalizations or physician visits or prescriptions for anxiety based on an algorithm used previously by the MCHP (http://mchp-appserv.cpe.umanitoba.ca/deliverablesList.html). Childhood anxiety was dichotomized (yes/no) based on having one or more hospitalizations, physician visits, **or** prescriptions by age 5 or having none of these indicators (see Table 1).

**Table 1:** Definitions of Dependent and Independent Variables.

Variable	Definition
**DEPENDENT VARIABLE**	
Childhood anxiety	Childhood anxiety was defined by a child meeting the following criteria at any time on or before the child’s fifth birthday (2003–2008/2004-2009):
	(a) one or more hospitalizations with a diagnosis for anxiety states, phobic disorders or obsessive-compulsive disorders, ICD-9-CM codes 300.0, 300.2, 300.3; ICD-10-CA codes F40, F40.0, F40.00, F40.01, F40.1, F40.2, F40.8, F40.9, F41, F41.0, F41.1, F41.3, F41.8, F41.9, F42, F42.0, F42.1, F42.2, F42.9, F43.0, F43.1, F43.2, F43.8, F43.9, F93, F93.0, F93.1, F93.2, F93.8, F93.9;
	(b) **OR** one or more physician visits with a diagnosis for anxiety disorders, ICD-9-CM code 300;
	(c) **OR** one or more prescriptions for anxiolytic medications, including chlordiazepoxide, diazepam, lorazepam, oxazepam, flurazepam, nitrazepam, alprazolam, clorazepate dipotassium, midazolam, chloral hydrate, triazolam, zopiclone, buspirone, bromazepam, clobazam, or temazepam.
**DEMOGRAPHIC**	
Maternal age	Age at delivery: <20, 20 to 34, ≥35 years
Maternal education	Completed high school versus not completed high school
Income assistance^a^	Receiving any form of income assistance during pregnancy
Income quintiles	Income quintile of neighborhood of residence based on Census data, categorized from Quintile 1 (lowest) to Quintile 5 (highest) (based on definition used previously by Manitoba Centre for Health Policy, http://mchp-appserv.cpe.umanitoba.ca/deliverablesList.html)
Relationship status	Lone parent versus married/partnered
**OBSTETRICAL**	
Parity	Multiparous vs primiparous
Type of delivery	Cesarean versus vaginal
Antepartum hemorrhage	A woman was categorized as having an antepartum haemorrhage if she had any of the following diagnostic codes recorded during pregnancy:
	ICD-9-CM Diagnosis Code 641;
	(b) **OR** ICD 10 (044, 45, 46);
	(c) **OR** ICD 9 641.
**PSYCHOSOCIAL**	
Social isolation	Mother’s response to nurse’s question (item C32) regarding social isolation e.g., Is the support enough for the mother/couple? If mother says she has no support, score ‘no’. Families First screening form defines social isolation as ‘lack of social support and/or isolation related to culture, language or geography’
Relationship distress	Mother’s response to nurse’s question (item C33) regarding relationship distress. Families First screening form defines relationship distress as ‘distress or conflict between parenting partners e.g. separations, frequent arguments’.
Prenatal psychological distress	A woman would be classified as having PRENATAL psychological distress if she met the following criteria during her pregnancy (2002-2003/2003-2004):
	(a) one or more hospitalizations with a diagnosis for depressive disorder, affective psychoses, neurotic depression or adjustment reaction, ICD-9-CM codes 296.2–296.8, 300.4, 309, 311; ICD-10-CA codes F31, F32, F33, F34.1, F38.0, F38.1, F41.2, F43.1, F43.2, F43.8, F53.0, F93.0;
	(b) **OR** one or more physician visits with a diagnosis for depressive disorder, affective psychoses or adjustment reaction, ICD-9-CM codes 296, 309, 311;
	(c) **OR** one or more hospitalizations with a diagnosis for anxiety disorders, ICD-9-CM code 300 (excluding 300.13, 300.14, 300.15); ICD-10-CA codes F32.0, F34.1, F40, F41, F44, F45.0, F45.1, F45.2, F48, F68.0, F99;
	(d) **OR** one or more prescriptions for an antidepressant or mood stabilizer, ATC codes N03AB02, N03AB52, N03AF01, N05AN01, N06A;
	(e) **OR** two or more physician visits with a diagnosis for anxiety disorders, ICD-9-CM code 300
Postnatal psychological distress	A woman would be classified as having postnatal psychological distress if she met at least one of the following criteria during the postpartum period (from birth to 12 months following birth):
	(a) one or more hospitalizations with a diagnosis for depressive disorder, affective psychoses, neurotic depression or adjustment reaction, ICD-9-CM codes 296.2–296.8, 300.4, 309, 311; ICD-10-CA codes F31, F32, F33, F34.1, F38.0, F38.1, F41.2, F43.1, F43.2, F43.8, F53.0, F93.0;
	(b) **OR** one or more physician visits with a diagnosis for depressive disorder, affective psychoses or adjustment reaction, ICD-9-CM codes 296, 309, 311;
	(c) **OR** one or more hospitalizations with a diagnosis for anxiety disorders, ICD-9-CM code 300 (excluding 300.13, 300.14, 300.15); ICD-10-CA codes F32.0, F34.1, F40, F41, F44, F45.0, F45.1, F45.2, F48, F68.0, F99;
	(d) **OR** one or more prescriptions for an antidepressant or mood stabilizer, ATC codes N03AB02, N03AB52, N03AF01, N05AN01, N06A;
	(e) **OR** two or more physician visits with a diagnosis for anxiety disorders, ICD-9-CM code 300
Psychological distress during early childhood period	A woman would be classified as having psychological distress during the early childhood period if she met at least one of the following criteria during the period encompassing the second year after the birth to her child’s fifth birthday (13 months to 5 years after birth):
	(a) one or more hospitalizations with a diagnosis for depressive disorder, affective psychoses, neurotic depression or adjustment reaction, ICD-9-CM codes 296.2–296.8, 300.4, 309, 311; ICD-10-CA codes F31, F32, F33, F34.1, F38.0, F38.1, F41.2, F43.1, F43.2, F43.8, F53.0, F93.0;
	(b) **OR** one or more physician visits with a diagnosis for depressive disorder, affective psychoses or adjustment reaction, ICD-9-CM codes 296, 309, 311;
	(c) **OR** one or more hospitalizations with a diagnosis for anxiety disorders, ICD-9-CM code 300 (excluding 300.13, 300.14, 300.15); ICD-10-CA codes F32.0, F34.1, F40, F41, F44, F45.0, F45.1, F45.2, F48, F68.0, F99;
	(d) **OR** one or more prescriptions for an antidepressant or mood stabilizer, ATC codes N03AB02, N03AB52, N03AF01, N05AN01, N06A;
	(e) **OR** three or more physician visits with a diagnosis for anxiety disorders, ICD-9-CM code 300
**MEDICAL**	
Diabetes	A woman was classified as having diabetes if she had:
	(a) 1+ hospitalizations with diagnosis code 250 (ICD-9 CM) or E10-E14 (ICD-10) in any diagnosis field over 3 years of data;
	(b) **OR** 2+ physician claims with diagnosis code 250 over 3 years of data;
	(c) **OR** 1+ prescriptions for diabetic drugs over 3 years of data*;
	(d) **OR** 1+ hospitalizations with gestational diabetes or diabetes mellitus in pregnancy (ICD 9 648.8 or ICD 10 024)**
Hypertension	A woman was classified as having hypertension if she had:
	at least one physician visit or one hospitalization related to hypertension prior to pregnancy (ICD-9-CM codes 401–405 OR ICD-10-CA codes I10-I13, I15);
	**OR** two or more prescriptions for hypertension drugs*;
	**OR** at least one physician visit or one hospitalization during pregnancy related to hypertension (ICD-9-CM code 642 OR ICD-10-CA codes 010-O16).**
**BEHAVIORAL**	
Prenatal substance use	Composite measure of alcohol use, drug use, **OR** smoking during pregnancy. Includes a ‘yes’ response to one or more of the following items on the Families First screen: i) ‘alcohol use by mother during pregnancy’; ii) ‘drug use by mother during pregnancy’; iii) ‘maternal smoking during pregnancy’.
**INFANT**	
Infant sex	Male versus female
Apgar score	5-minute Apgar score ≤7
Preterm birth	Delivery at <37 weeks
Small for gestational age	birth weight <10^th^ percentile for gestational age and sex
Breastfeeding initiation	Initiation in hospital: yes versus no

**Note.** We hypothesized that low maternal education, prenatal substance use, social isolation, and relationship distress could adversely impact child anxiety. These variables were unavailable in the main administrative databases. Thus, we abstracted them from the Families First screen data that are collected by Healthy Child Manitoba. The Families First screen is conducted by public health nurses during a home visit scheduled shortly after delivery and is intended to identify biological and social risk factors as well as provide education and links to community support (http://www.gov.mb.ca/healthychild/familiesfirst/). Some data (e.g., education) on the Families First screen are missing if they were not collected at the time of the public health nurse visit.

^a^Manitoba Jobs and the Economy currently administers the income assistance data; formerly Manitoba Family Services.

*See list of drugs used in Manitoba RHA Indicators Atlas 2009.

**Based on previous definitions used by the Manitoba Centre for Health Policy http://mchp-appserv.cpe.umanitoba.ca/deliverablesList.html.

Definitions of the independent variables are in Table 1, and were categorized as prenatal if they were measured from conception to birth, postnatal if measured from birth to 12 months post-delivery, and early childhood if measured from 13 months to the child’s fifth birthday.

### Analysis

Descriptive statistics (means, standard deviations, percentages) were generated. Two-tailed tests were conducted, and statistical significance for all analyses was defined as p<0.05. Univariable analyses were conducted to produce unadjusted odds ratios (UORs) of the association between each independent variable and the outcome. Variables in the multivariable models were adjusted for each other, reported as adjusted odds ratios (AORs) and 95% confidence intervals (CI). We assessed multicollinearity among the independent variables based on variation inflation factors (VIFs) and tolerance levels (TLs), with multicollinearity defined as VIFs >2.5 and TLs <0.40 [15]. Because data missing from the Families First screen may not be random, we reported proportions of missing data for these variables and included the missing category in the regression analyses. The analyses were conducted using SAS Version 9.1.

The study was approved by the Health Research Ethics Board (University of Manitoba) and the provincial Health Information Privacy Committee. Because the study utilized anonymized and de-identified administrative data, individual-level consent from participants was not required.

## Results

The sample consisted of 19316 women. Of the 26432 women who delivered singleton infants in 2003 and 2004, 19770 had a completed Family First screen with 454 excluded because the child was not living with the mother at age 5 (died or in foster care). Missing data comprised 2.5% with final regression models based on a sample size of 18836 women/child dyads. The majority of women were 20–34 years of age (M = 28.2 years, SD = 3.93) (Table 2). Approximately 18% of women had not completed high school and 19% received income assistance during pregnancy. Among the infants in the sample, 6.1% were preterm, 6.9% were small for gestational age, and 3.4% had an Apgar score of ≤7 (Table 2).

**Table 2:** Prenatal, Postnatal, and Early Childhood Predictors of Childhood Anxiety in a Population-based Sample (Manitoba, Canada).

Independent variable	Childhood anxiety	No childhood anxiety	Unadjusted Model		Adjusted Model	
	(n = 591)	(n = 18725)			(N = 18836)	
	N (%)	N (%)				
	N (%)	N (%)	UOR^a^	95% CI^a^	AOR^a^	95% CI
Maternal age at birth^a^						
<20 years	32 (5.4)	1105 (5.9)	.90	.63–1.29	**.66**	**.43–.97**
20–34 years	485 (82.1)	15063 (80.4)	1.00	(reference)	1.00	(reference)
≥35 years	74 (12.5)	2557 (13.7)	.90	.70–1.15	.96	.74–1.24
Completed high school						
No	121 (20.5)	3315 (17.7)	1.16	.95–1.43	1.18	.93–1.51
Yes	425 (71.9)	13533 (72.3)	1.00	(reference)	1.00	(reference)
Missing	45 (7.6)	1877 (10.0)	.76	.56–1.04	**.67**	**.47–.96**
On income assistance (during pregnancy)^a^						
Yes	127 (21.5)	3511 (18.8)	1.19	.97–1.45	1.00	.76–1.32
No	464 (78.5)	15214 (81.2)	1.00	(reference)	1.00	(reference)
Neighborhood income quintile						
Quintile 1 (lowest)	138 (23.5)	3795 (20.3)	1.20	.93–1.55	1.16	.88–1.53
Quintile 2	129 (21.9)	3754 (20.0)	1.13	.87–1.47	1.08	.83–1.42
Quintile 3	103 (17.4)	3807 (20.3)	.89	.68–1.18	.87	.66–1.15
Quintile 4	114 (19.4)	3860 (20.6)	.97	.74–1.27	.93	.71–1.22
Quintile 5 (highest)	105 (17.8)	3461 (18.5)	1.00	(reference)	1.00	(reference)
Relationship status						
Lone parent	73 (12.4)	1884 (10.1)	.79	.62–1.02	1.14	.83–1.56
Married/partnered	499 (84.4)	16239 (86.7)	1.00	(reference)	1.00	(reference)
Unknown	19 (3.2)	602 (3.2)	.97	.61–1.55	.93	.54–1.60
Parity^a^						
Primiparous	280 (47.4)	7585 (40.5)	1.00	(reference)	1.00	(reference)
Multiparous	311 (52.6)	11125 (59.4)	.76	.64–.89	**.73**	**.61–.87**
Cesarean delivery						
Yes	126 (21.3)	3889 (20.1)	1.03	.85–1.26	1.004	.82–1.23
No	465 (78.7)	14836 (79.2)	1.00	(reference)	1.00	(reference)
Antepartum hemorrhage						
Yes	35 (5.9)	950 (5.1)	1.18	.83–1.67	1.16	.81–1.66
No	556 (94.1)	17775 (94.9)	1.00	(reference)	1.00	(reference)
Social isolation						
Yes	28 (4.7)	736 (3.9)	1.24	.84–1.83	1.09	.72–1.65
No	501 (84.8)	16308 (87.1)	1.00	(reference)	1.00	(reference)
Unknown	62 (10.5)	1681 (9.0)	1.20	.92–1.57	1.42	.95–2.11
Relationship distress						
Yes	32 (5.4)	809 (4.2)	1.29	.89–1.85	1.04	.69–1.58
No	460 (77.8)	14940 (80.0)	1.00	(reference)	1.00	(reference)
Unknown	99 (16.8)	2976 (15.8)	1.08	.87–1.35	1.04	.77–1.41
Prenatal psychological distress: Conception to birth						
Yes	64 (10.8)	1337 (7.1)	1.58	1.21–2.06	1.13	.84–1.52
No	527 (89.2)	17388 (92.9)	1.00	(reference)	1.00	(reference)
Postnatal psychological distress: Birth to 12 months						
Yes	116 (19.6)	2418 (12.9)	1.65	1.34–2.03	**1.28**	**1.001–1.64**
No	475 (80.4)	16307 (87.1)	1.00	(reference)	1.00	(reference)
Psychological distress in early childhood: 13 months to 5^th^ birthday						
Yes	236 (40.0)	5480 (29.3)	1.61	1.36–1.90	**1.44**	**1.19–1.75**
No	355 (60.0)	13245 (70.7)	1.00	(reference)	1.00	(reference)
Diabetes (before or during pregnancy)						
Yes	38 (6.4)	895 (4.8)	1.37	.98–1.92	1.29	.90–1.83
No	553 (93.6)	17830 (95.2)	1.00	(reference)	1.00	(reference)
Hypertension (before or during pregnancy)						
Yes	65 (11.0)	1859 (9.9)	1.12	.86–1.46	1.05	.80–1.37
No	526 (89.0)	16866 (90.1)	1.00	(reference)	1.00	(reference)
Prenatal substance use						
Yes	154 (26.1)	4604 (24.6)	1.08	.90–1.31	.98	.80–1.22
No	414 (70.1)	13409 (71.6)	1.00	(reference)	1.00	(reference)
Unknown	23 (3.8)	712 (3.8)	1.05	.68–1.60	.96	.56–1.63
Infant sex^a^						
Boy	303 (51.3)	9532 (50.9)	1.01	.86–1.19	.98	.83–1.15
Girl	288 (48.7)	9153 (48.9)	1.00	(reference)	1.00	(reference)
5-minute Apgar score ≤7^a^						
Yes	32 (5.4)	603 (3.2)	1.71	1.19–2.47	**1.76**	**1.20–2.58**
No	552 (93.4)	17781 (95.0)	1.00	(reference)	1.00	(reference)
Preterm (gestational age <37 weeks)^a^						
Yes	29 (4.9)	1133 (6.1)	.80	.55–1.17	**.67**	**.45–.999**
No	560 (94.8)	17477 (93.3)	1.00	(reference)	1.00	(reference)
Small for gestational age (<10^th^ percentile)^a^						
Yes	46 (7.8)	1269 (6.8)	1.16	.85–1.57	1.06	.77–1.45
No	542 (91.7)	17306 (92.4)	1.00	(reference)	1.00	(reference)
Breastfeeding initiation^a^						
No	78 (13.2)	2523 (13.5)	1.03	.81–1.31	1.09	.84–1.40
Yes	506 (85.6)	15871 (84.8)	1.00	(reference)	1.00	(reference)

Note: Variables in final multivariable model adjusted for all other variables in model. UOR = Unadjusted Odds Ratios; AOR = Adjusted Odds Ratios; CI = Confidence Interval.

^a^Missing data: maternal age = 153; income quintile = 50; parity = 15; child sex = 40; Apgar score = 338; preterm birth = 117; small for gestational age = 153; breastfeeding initiation = 338.

Seven percent of women experienced prenatal psychological distress with almost twice as many (13%) having postnatal distress from birth to 12 months. Almost 30% of women met criteria for psychological distress from 13 months to their child’s fifth birthday (Table 2). The period prevalence of childhood anxiety (birth to age 5) was 3.1% (n = 591). Among the children with anxiety, 73.8% (n = 436) had one or more physician visits for anxiety, 24.9% (n = 147) had a prescription for an anxiolytic, and 1.3% (n = 8) had both. No children in the sample were hospitalized for anxiety.

UORs and the multivariable regression models are in Table 2. No multicollinearity was detected among the independent variables (TIFs 0.57–0.99; VIFs 1.00–1.72). Young maternal age (<20 years) was associated with significantly lower odds of childhood anxiety compared to women aged 20–34 years. The only significant obstetrical factor related to childhood anxiety was parity, where children of multiparous mothers were 27% less likely to develop childhood anxiety. Among the psychosocial factors, maternal psychological distress from birth to 12 months, and 13 months to 5 years were independently associated with increased odds of childhood anxiety. Prenatal psychological distress was significantly related to childhood anxiety in unadjusted analyses, but not in the final multivariable model. None of the medical or behavioral variables were significant. While preterm children were less likely to develop childhood anxiety, children with Apgar scores of ≤7 had almost twice the odds of being affected.

## Discussion

This study is the first to report on the association between multiple prenatal, postnatal, and early life predictors and childhood anxiety up to age 5. The results of this study indicated that maternal psychological distress experienced from birth to 12 months and 13 months to 5 years, and having an Apgar score ≤7 increased the odds of childhood anxiety by age 5, whereas young maternal age, multiparity, and preterm birth were associated with reduced odds. Our study extends the small body of evidence of predictors of anxiety in children and addresses key limitations by studying very young children, using data from a large, population-based database, and measuring childhood anxiety objectively through physician visits and prescription medications.

This study provides evidence that maternal postnatal psychological distress is a significant predictor of childhood anxiety after controlling for distress in the prenatal and early childhood periods. We found no other studies that examined this association. Some studies reported associations with internalizing behavior (withdrawal, inhibition, shyness, anxiety, depression, somatic complaints) in young children [16], after adjusting [9] and not adjusting for [17,18] prenatal distress. In contrast to our study, using birth cohort data from the Avon Longitudinal Study of Parents and Children (ALSPAC), O’Connor et al. did not find an association between postnatal depression or anxiety and internalizing behavior once controlled for prenatal depression and anxiety [19]. We posit that these divergent findings may relate to the degree of severity of depression and anxiety. Given that we measured maternal postnatal distress by physician diagnosis, hospitalization, and prescriptions for anxiety and depression, it is likely that our cohort of women experienced more severe symptoms than the ALSPAC cohort. Indeed, a recent study indicated that the greatest impact of maternal distress on child behavioural-emotional difficulties was found in women with greater symptom severity, compared with women with lower severity [20].

Our study addresses a major limitation of existent evidence regarding the association between maternal distress in early childhood and childhood anxiety by controlling for prenatal or postnatal maternal psychological distress and thus isolating the timing of distress. Our findings replicated those of Fihrer et al. who reported postnatal and ‘current’ distress as independent predictors of internalizing behavior in a small sample (N = 75) of 6 to 8 year olds [18]. The mechanisms underlying the association between maternal mental health problems in early childhood and childhood anxiety are poorly understood [3], although child characteristics (cognitive biases [21], difficulty with emotions, poor self-concept [22], high behavioral inhibition [10], low external locus of control [22]) and controlling, overprotective parenting styles [12] are central to current hypotheses. Recent studies provide support for an epigenetic effect, where poor quality parenting results in DNA methylation, impacting gene expression in a manner that increases children’s vulnerability to suboptimal mental health [14]. Additionally, others have reported a bi-directional association between maternal distress in early childhood and preschooler behaviour problems, highlighting the importance of considering the impact of child difficulties on maternal distress [23,24].

We found no other studies linking Apgar scores and anxiety in young children, although one study reported an association between low Apgar scores and internalizing symptoms in adolescents [25]. Given that Apgar scores are a measure of autonomic transition in response to birth stress, it is plausible that Apgar scores ≤7 reflect disrupted neuroregulatory systems [25]. Based on the developmental theory of biological sensitivity to context, the unfavourable intrauterine environment recalibrates the fetal stress system, creating a neurobiological susceptibility to stressors that may be exhibited as child anxiety [26].

Similar to our study, others have reported a lack of relationship between childhood anxiety and sociodemographic factors (child sex [9,17,18]; socioeconomic status [10]; ethnicity [9]), obstetrical complications [9], breastfeeding [9], and risky behavior [9].

Evidence for an association between maternal age and child anxiety is inconsistent, although understudied. While some have not found associations with internalizing behavior [9], others report impaired socio-emotional development in children of young mothers [27]. Similar to our study, O’Connor et al. found a protective relationship between age and internalizing/externalizing behavior at age 4 [19]. It may be that the barriers to access that young mothers face with respect to their own mental healthcare and lower treatment engagement may impact their decision to seek mental healthcare for their child, and thus the lower prevalence reflects reduced access [23,28].

The reduced odds of anxiety in children of multiparous mothers that we observed is similar to Robinson’s study that reported a 23% decrease in the odds of internalizing problems at age 5 in children with more siblings (AOR .77, 95% CI .63–.95) [9]. Children with siblings may benefit from greater social support derived from sibling relationships, particularly since separation and social anxiety start to peak at ages 3 to 6 years [3]. Siblings may also play a role in regulating child cognitions. Alternatively, women with other children may be less apt to identify anxiety symptoms (more relaxed/less reactive than first-time mothers), distracted by other children, or too busy to seek treatment for the child.

Our finding regarding the reduced odds of anxiety in children born preterm adds to the equivocal evidence regarding the relationship between gestational age and child emotional problems. While some have reported increased risk [9,25] and no association [4], others have found that prematurity is protective of emotional difficulties [19]. With respect to anxiety in particular, studies that have distinguished between neurodevelopmental conditions (autism, attention deficit hyperactivity disorder, developmental delay, learning disability) and psychiatric morbidity have found no additional risk of anxiety between term and preterm children. For example, data from the National Survey of Children’s Health (N = 95,677) suggest that increased risk of anxiety is found only among preterm children with comorbid neurodevelopmental conditions [29]. There is some suggestion from meta-analyses that the relationship between prematurity and emotional problems also varies by the era in which the preterm infant was born, with early studies (<1990) showing an increased risk and later studies (>1998) showing no association [30]. It is likely that biological and environmental pathways operate in concert, where impaired hypothalamic-pituitary-adrenal-axis functioning, in combination with risky extrauterine factors increase the risk of anxiety [4]. Thus, the reduced likelihood of childhood anxiety in preterm infants that we report may be related to the: 1) protective influence of a reduced length of time exposed to a compromised, suboptimal intrauterine environment (fetal programming hypothesis) [14]; in this case, reduced time in-utero may decrease exposure to pathological events, as described in the fetus-at-risk paradigm where the benefit of preterm delivery in terms of ameliorating risk of adverse fetal and neonatal outcomes may exceed that of term delivery [31]; 2) attenuation of risk as a result of early detection and treatment of anxiety symptoms during routine developmental follow-up of preterm infants; or 3) advanced neonatal care that improved neurodevelopmental outcomes in our cohort born 2003–2004 [30].

### Strengths and Limitations

A strength of this study is the inclusion of multiple early life factors from pregnancy to age 5. The population-based sample enhances the study’s generalizability. However, some limitations are inherent in the use of administrative data. We were unable to explore some predictors that others have demonstrated as important (parenting, peers, child temperament [3], child cognitions [21]). Similarly, given that cognitive behavior therapy is commonly used to treat mild/moderate childhood anxiety [24], but not reported in administrative databases, and anxiety in children is often unrecognized and untreated [3], the children we studied likely represent those with greater symptom severity. As such, the findings may not be generalizable to children with less severe anxiety. However, a recent study (N = 1,085) reported that 4-year old children of women with both severe and sub-clinical depressive symptoms from pregnancy to 4-years postpartum were more likely to experience emotional difficulties (23% vs 19%, respectively) compared to women with no symptoms (7%), [20] suggesting that sub-clinical symptoms can also be associated with emotional problems in preschoolers. The prevalence rate of anxiety may have been influenced by: 1) measuring childhood anxiety by a single diagnostic code, with the possibility that transient cases may be erroneously classified as ‘childhood anxiety’; 2) anxiolytics that are prescribed for disorders other than anxiety; 3) not capturing children who were prescribed antidepressants (SSRI’s) for anxiety [26]; and under-recognition or false positive diagnoses by physicians [32]. While using medication, diagnosis, and hospitalization to measure maternal distress was unique to this field, databases do not capture women receiving non-pharmacological treatment. Thus, our sample may comprise women with greater severity who sought treatment, and our findings may not be generalizable to women with less severe symptoms. In this regard, women with a diagnosis or treatment history for anxiety or depression may be more sensitive to detecting anxiety in their children and thus seeking healthcare; however, given that child anxiety was measured objectively by physician visits and prescriptions (versus maternal report), we do not anticipate that maternal heightened sensitivity contributed to a spurious finding regarding the association between maternal psychological distress and child anxiety. Finally, First Nations women living on reserves were not involved in the Families First program, and therefore the findings are not generalizable to this group.

## Conclusion

Identifying risk factors for childhood anxiety is a key step to improving detection, prevention, and early intervention [3]. These findings can be used to improve detection of anxiety in children by informing the development of a broader approach to psychosocial assessment involving the integration of risk factors with symptom-based screening tools. Evidence of the importance of maternal psychological distress from birth to age 5 also supports recommendations by national pediatric associations to conduct maternal mental health screening during well-child visits [33]. Future research should aim to disentangle the early life influences on childhood anxiety occurring in the prenatal, postnatal, and early childhood periods, understand the underlying mechanisms, and evaluate the effectiveness of prenatal and postnatal intervention on childhood anxiety.
